# Single gene-based distinction of individual microbial genomes from a mixed population of microbial cells

**DOI:** 10.3389/fmicb.2015.00195

**Published:** 2015-03-11

**Authors:** Manu V. Tamminen, Marko P. J. Virta

**Affiliations:** ^1^Department of Food and Environmental Sciences, University of HelsinkiHelsinki, Finland

**Keywords:** multiple displacement amplification, *in situ* PCR, fluorescent hybridization, flow cytometry

## Abstract

Recent progress in environmental microbiology has revealed vast populations of microbes in any given habitat that cannot be detected by conventional culturing strategies. The use of sensitive genetic detection methods such as CARD-FISH and *in situ* PCR have been limited by the cell wall permeabilization requirement that cannot be performed similarly on all cell types without lysing some and leaving some nonpermeabilized. Furthermore, the detection of low copy targets such as genes present in single copies in the microbial genomes, has remained problematic. We describe an emulsion-based procedure to trap individual microbial cells into picoliter-volume polyacrylamide droplets that provide a rigid support for genetic material and therefore allow complete degradation of cellular material to expose the individual genomes. The polyacrylamide droplets are subsequently converted into picoliter-scale reactors for genome amplification. The amplified genomes are labeled based on the presence of a target gene and differentiated from those that do not contain the gene by flow cytometry. Using the *Escherichia coli* strains XL1 and MC1061, which differ with respect to the presence (XL1), or absence (MC1061) of a single copy of a tetracycline resistance gene per genome, we demonstrate that XL1 genomes present at 0.1% of MC1061 genomes can be differentiated using this method. Using a spiked sediment microbial sample, we demonstrate that the method is applicable to highly complex environmental microbial communities as a target gene-based screen for individual microbes. The method provides a novel tool for enumerating functional cell populations in complex microbial communities. We envision that the method could be optimized for fluorescence-activated cell sorting to enrich genetic material of interest from complex environmental samples.

## Introduction

The immense diversity of microbes in the environment is a serious obstacle to determine the presence of different genetic properties of individual microbial cells. Methods such as CARD-FISH and *in situ*-PCR that allow detection of low copy genetic targets in individual cells are limited by the different properties of bacterial cell surfaces that have to be permeabilized to allow diffusion of reagents into the cells without lysing them (Hodson et al., 1995; Kubota et al., 2008). Traditional FISH methods that rely on small oligonucleotide probes are less hampered by the permeabilization requirements but on the other hand are limited to high copy targets such as ribosomal RNA (De Long et al., 1989; Amann et al., 1995). Combination of these two benefits to permit the detection of low copy genomic targets in diverse microbial populations has remained problematic.

Multiple displacement amplification (MDA) reaction has recently been developed for unspecific amplification of very low amounts of target DNA (Dean et al., 2002). The reaction has been shown to be capable of amplification of single-genomes into microgram amounts of DNA (Marcy et al., 2007; Stepanauskas and Sieracki, 2007) and has also been shown to function in nanoliter reaction volumes (Marcy et al., 2007). Until now, the applicability of MDA to amplify rare genomic targets to assist FISH detection has not been demonstrated.

Here, we present a technique that permits an unbiased genome exposure of individual microbial cells by trapping them individually into rigid polyacrylamide droplets that support their genomes even after cell material has been degraded. The droplets are subsequently converted into picoliter-volume agarose reactors (picoreactors) and used for single-genome MDA reaction in an emulsion. A polymerase chain reaction (PCR)-based approach is used to fluorescently label the picoreactors containing genomic DNA that contains the target gene. Labeled and unlabeled picoreactors are differentiated using flow cytometry, which is capable of distinguishing target populations that constitute as little as 0.1% of the total microbial genome sample. In this proof-of-concept study, we differentiate two *Escherichia coli* strains that differ with respect to the presence (*E. coli* XL1; Keasling et al., 1991) or absence (*E. coli* MC1061) of a singe copy per genome of a tetracycline resistance gene. We furthermore demonstrate the applicability of the technique to highly complex environmental samples by differentiating *E. coli* XL1 genomes from a mixture with diverse microbial cells extracted from marine sediment. The technique could be optimized for fluorescence-activated cell sorting to screen and capture genetic material of interest from complex environmental microbial communities.

## Materials and Methods

### Bacterial Immobilization in Polyacrylamide Beads

*Escherichia coli* XL1 and MC1061 cells were cultivated in LB medium until reaching stationary phase, fixed for 2 h with 2% paraformaldehyde (Merck, Darmstadt, Germany) in PBS and stored in aqueous 50% ethanol at -20°C until use. The cell density was determined using an LSR II flow cytometer (Becton–Dickinson, Franklin Lakes, NJ, USA). The microbial cells were extracted from the marine sediment using a previously published method (Kallmeyer et al., 2008) and fixed as described above. The cell density was determined using a Zeiss Axiovert 200 M fluorescence microscope. The fixed cells were filtered through a 5-μm nitrocellulose filter (Millipore, Cork, Ireland) and suspended in 250 μl of acrylic suspension containing 20% acrylamide (Sigma, St. Louis, MO, USA), 0.27% *N*,*N′*-bisacryloylcystamine (Sigma, St. Louis, MO, USA) and 0.5% ammonium persulphate (Sigma, St. Louis, MO, USA). The suspension was mixed as described in (Williams et al., 2006) by drop-wise addition using an ordinary pipette (5 s between drops) into 400 μl of mineral oil containing 4.5% Span 80 (Sigma–Aldrich, St. Louis, MO, USA), 0.4% Tween 80 (Sigma, St. Louis, MO, USA) and 0.05% Triton X-100 (Applichem, Darmstadt, Germany) with stirring at 1,000 rpm using an IKA Yellowline MSH basic laboratory stirrer. After addition of the acrylic phase, TEMED (Sigma, St. Louis, MO, USA) was added on top of the emulsion to a concentration of 3.8% (25 μl of TEMED). Stirring was continued for 1 h to allow complete polymerization. After polymerization, the emulsion was disrupted with 2 ml of water-saturated diethylether (Sigma, St. Louis, MO, USA). The polyacrylamide droplets were washed by suspending into 2 ml of sterile water and recovered by centrifugation for 30 s at 8,000*g*. Washes were repeated until all traces of oil and ether had disappeared.

### Cell Lysis

Polyacrylamide droplets (200 μl) were suspended in 800 μl of 1x PCR buffer (Fermentas), after which 13 U of proteinase K (Sigma–Aldrich, St. Louis, MO, USA) was added and the suspension was incubated at 37°C for 1 h. After heat-inactivating proteinase K at 90°C for 1 min, 20 μg of lysozyme (Sigma, St. Louis, MO, USA) was added and the suspension was incubated at 37°C for 1 h. Finally, 26 U of proteinase K was added and the suspension was incubated for 5 h at 55°C. Enzymes were inactivated by heating at 90°C for 5 min.

### Forming Agarose Shells on Polyacrylamide Droplets

A 20-μl aliquot of polyacrylamide droplets, prepared as described above, was mixed with 200 μl of agarose IV solution (Amersco, OH, USA) to yield a 1% agarose suspension. The agarose suspension was kept at 45°C to prevent solidifying. The temperature-equilibrated agarose suspension was mixed drop-wise (5 s between drops) into 1 ml of warm (50°C) emulsion oil using an ordinary pipette, then stirred at 1,000 rpm for 5 min and mixed with 4 ml of ice-cold emulsion oil. After holding on ice for 15 min, the suspension was divided into 2-ml microcentrifuge tubes (Eppendorf, Hamburg, Germany), and excess emulsion oil was removed by centrifuging for 10 min at 12,000*g* and discarding the upper layer. The remaining emulsion oil was dissolved in water-saturated diethylether, and the picoreactors were washed as described above. Finally, agarose picoreactors were filtered through a cell strainer with a 40 μm mesh (BD Falcon, Franklin Lakes, NJ, USA).

### MDA Reaction and Second Polyacrylamide Layer Formation

To allow subsequent MDA reactions, the polyacrylamide matrix was dissolved by mixing 38 μl of the agarose picoreactors in 150 μl of a solution containing 87 μl of Repli-g Buffer (Qiagen, Hilden, Germany), 10 μl of Repli-g Φ29 polymerase (Qiagen, Hilden, Germany), 1 μg/μl BSA (Roche, Mannheim, Germany) and 6.7 μM dithiothreitol (Qiagen, Hilden, Germany). The reagents, with the exception of the Repli-g Buffer, were sterilized under UV light for 10 min prior to use. The solution was mixed with 400 μl of emulsion oil and incubated overnight at 30°C. The emulsion oil was dissolved in water-saturated diethylether, and the droplets were washed as described above. The agarose picoreactor droplets were suspended in an acrylic suspension prepared as above, but also containing an acrydite-modified forward primer (5′-Acrydite-TAC GTG AAT TTA TTG CTT CGG-3′; IDT, Berchem, Belgium) at a final concentration of 1.0 μM in a total volume of 250 μl. The suspension was mixed drop-wise (5 s between drops) into emulsion oil while stirring at 1,000 rpm. After addition of the acrylic phase, TEMED was added on top of the emulsion to a concentration of 3.8% (25 μl of TEMED). Stirring was continued for 1 h to allow complete polymerization. After polyacrylamide polymerization, the emulsion oil was dissolved in water-saturated diethylether, and the polyacrylamide-agarose picoreactors were washed as described above.

### Emulsion PCR

Polyacrylamide-agarose picoreactors (148 μl) were mixed into 1x Hot Start PCR Buffer containing 2.0 mM MgCl_2_ (Fermentas, St. Leon-Rot, Germany), 0.2 mM dNTP mixture (0.2 mM each, dTTP substituted with dUTP), 0.01 μM forward primer (5′-TAC GTG AAT TTA TTG CTT CGG-3′), 2.5 μM reverse primer (5′-ATA CAG CAT CCA AAG CGC AC-3′; Oligomer, Helsinki, Finland), and 50 U of Maxima Hot Start Taq Polymerase (Fermentas, St. Leon-Rot, Germany) in a total reaction volume of 200 μl. The PCR mixture was added drop-wise to the PCR emulsion oil as described in (Williams et al., 2006) with constant stirring (1,000 rpm), and then allowed to continue mixing for an additional 5 min. The mixture was then divided into 50-μl aliquots on PCR strips, overlaid with 50 μl of mineral oil and subjected to PCR thermal cycling. Cycling conditions were 5 min at 95°C, 25 cycles of 1 min at 95°C, 1 min at 55°C and 2 min at 72°C, with a final extension of 10 min at 72°C. After PCR, the emulsion was pooled in 2 ml microcentrifuge tubes (Eppendorf, Hamburg, Germany), and the excess emulsion oil was removed by centrifuging for 10 min at 12,000*g* and discarding the upper layer. The remaining emulsion oil was dissolved in water-saturated diethylether, and the droplets were washed as described above.

### Picoreactor Labeling

After PCR, polyacrylamide-agarose picoreactors were mixed with 400 μl of hybridization buffer containing 0.9 M NaCl (Sigma–Aldrich, St. Louis, MO, USA), 20 mM Tris-HCl pH 8 (Sigma–Aldrich, St. Louis, MO, USA), 0.02% sodium dodecyl sulfate (Sigma, St. Louis, MO, USA) and 20% formamide (Applichem, Darmstadt, Germany). After adding a 5′-Cy5-labeled red fluorescent probe (5′-GCG CCT ATT AAT GAC AAC AA-3′) to a concentration of 5 pM, the probe-picoreactor mixture was heated to 100°C for 2 min and then hybridized by incubating at 46°C for 1 h. The hybridization mixture was washed by first incubating with hybridization buffer for 20 min at 48°C and then incubating with hybridization buffer without formamide for 20 min at 48°C; thereafter, the washed picoreactors were suspended in 400 μl of hybridization buffer without formamide. Between washing steps, picoreactors were collected by centrifuging mixtures for 30 s at 8,000*g*. A SYBR Green stock solution was added to a final concentration of 0.13% to give a green fluorescent signal to DNA-containing polyacrylamide-agarose picoreactors.

### Flow Cytometry and Microscopy

The polyacrylamide-agarose picoreactors were analyzed based on green (fluorescein isothiocyanate, FITC) and red (allophycocyanin, APC) fluorescence using an LSR II flow cytometer (Becton-Dickinson, Franklin Lakes, NJ, USA). Successfully assembled picoreactors were separated from empty agarose droplets by increased side scatter value. The labeled picoreactors containing amplified *E. coli* XL1 genomic DNA (with the tetracycline resistance gene) could be differentiated from those containing amplified *E. coli* MC1061 genomic DNA (without the tetracycline resistance gene) by their increased fluorescence. Micrographs for were taken using SP5 (**Figures 2A,B**) and SP2 (**Figure 4**) confocal microscopes using objective HCX PL APO 63x/1.2W Corr, 0.17 CS (Leica, Wetzlar, Germany). The software used was LCS 2.61.1537 for SP2 and LAS AF 2.2.0 build 4765 for SP5. The differential interference contrast and confocal micrographs were combined, and the scales were drawn using ImageJ (Abramoff et al., 2004).

## Results

### Bacterial Trapping in Polyacrylamide Droplets and Genome Exposure

**FIGURE 1:**
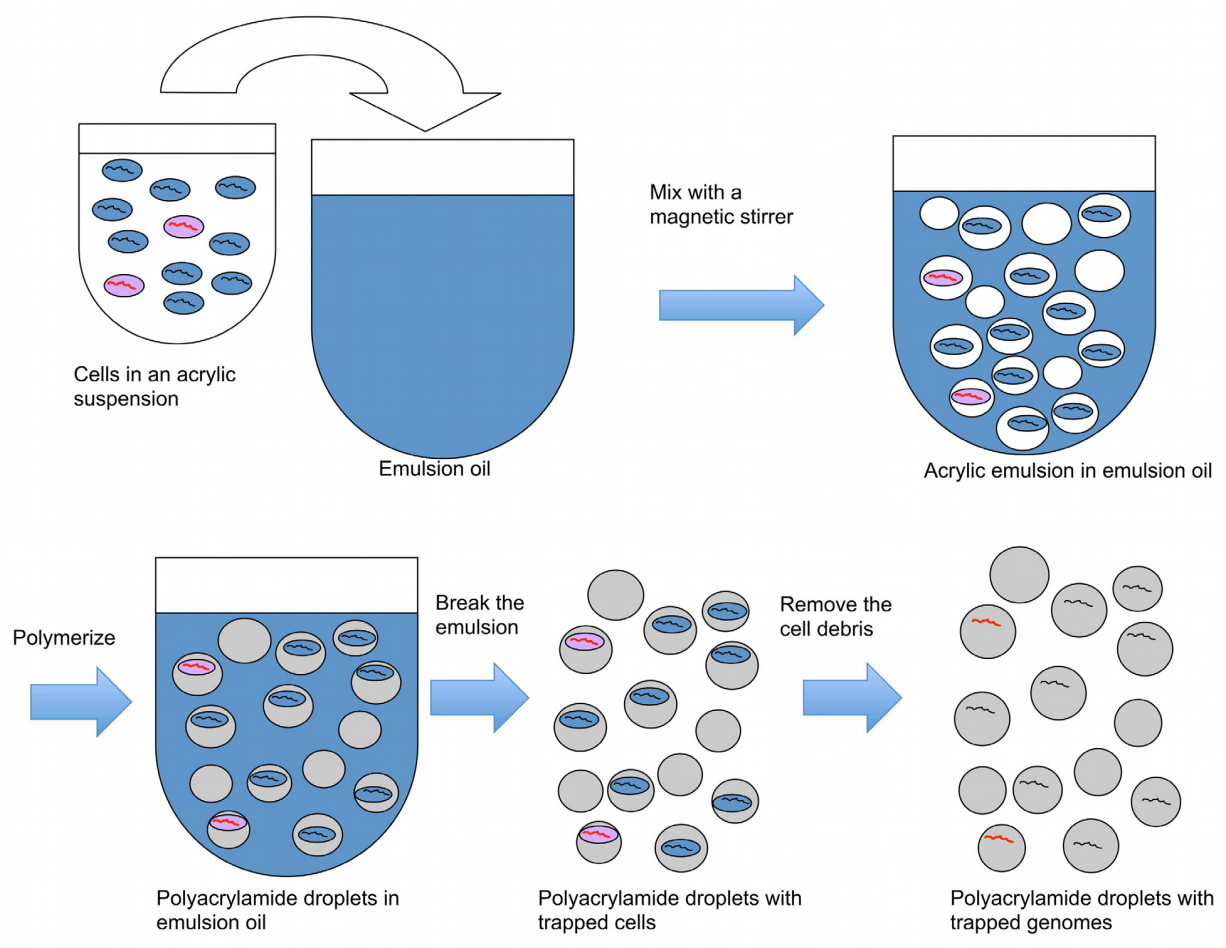
**A procedure to create polyacrylamide droplets containing individual genomes.** Cells in acrylic suspension are mixed into emulsion oil. The emulsion droplets polymerize to yield polyacrylamide droplets containing single cells. The emulsion is broken and the cells in the polyacrylamide droplets are treated enzymatically to destroy cell walls, membranes, and protein components, and expose genomic DNA. Black lines represent genomes without the target gene, and red lines represent genomes with the target gene.

**FIGURE 2:**
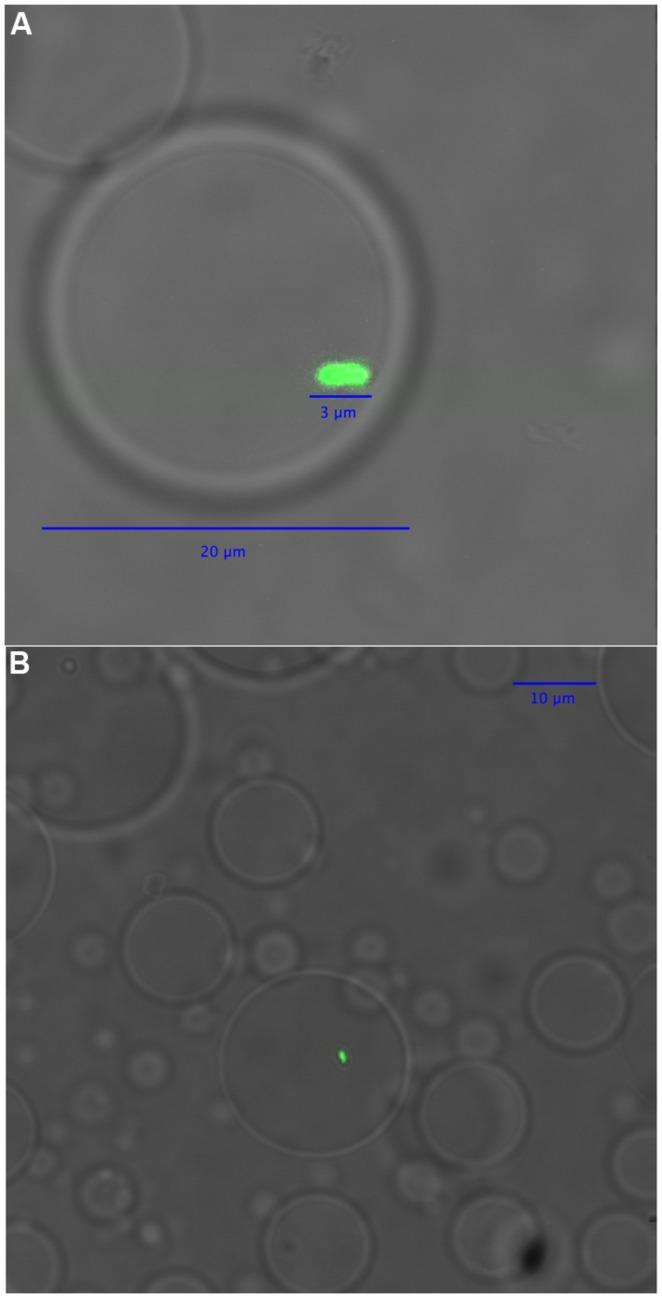
**A differential interference contrast/confocal micrograph of **(A)** a polyacrylamide droplet containing an *Escherichia coli* XL1 genome after cell lysis, and **(B)** several polyacrylamide droplets, one of which contains an *E. coli* XL1 genome.** The green fluorescence of SYBR Green dye is used to visualize DNA.

In the first part of the technique, acrylamide is polymerized on microbial cells, trapping cells in emulsion droplets (**Figure 1**). In this state, cell walls and other cellular components can be enzymatically removed, allowing microbial genomes to be exposed to further reactions. The exposed genomes do not diffuse into the surrounding liquid because they are covered and supported by the polyacrylamide matrix (**Figures 1** and **2A**). The polyacrylamide is prepared using a special cross-linker that is dissolved by the mild reducing conditions of the subsequent MDA reaction.

When a dilute cell suspension is mixed into an emulsion, the number of cells in each emulsion droplet follows a Poisson distribution with an average number of cells per droplet of less than one. Empty emulsion droplets are most common, followed by droplets containing one cell; the frequency of droplets with more than one cell is very low compared with those with one cell. Therefore, mixing cells in an acrylamide suspension into an oil emulsion for polymerization is an efficient way to create polyacrylamide droplets that capture single cells. The diameter of the polyacrylamide droplets typically ranged from about 5–30 μm with most droplets having a diameter around 10 μm (**Figure 2B**). The corresponding volume of the droplets therefore ranged from 0.07 to 14 picoliters, calculated using the formula *V* = 4/3πr^3^.

### Creating Picoreactors and Performing Emulsion MDA

**FIGURE 3:**
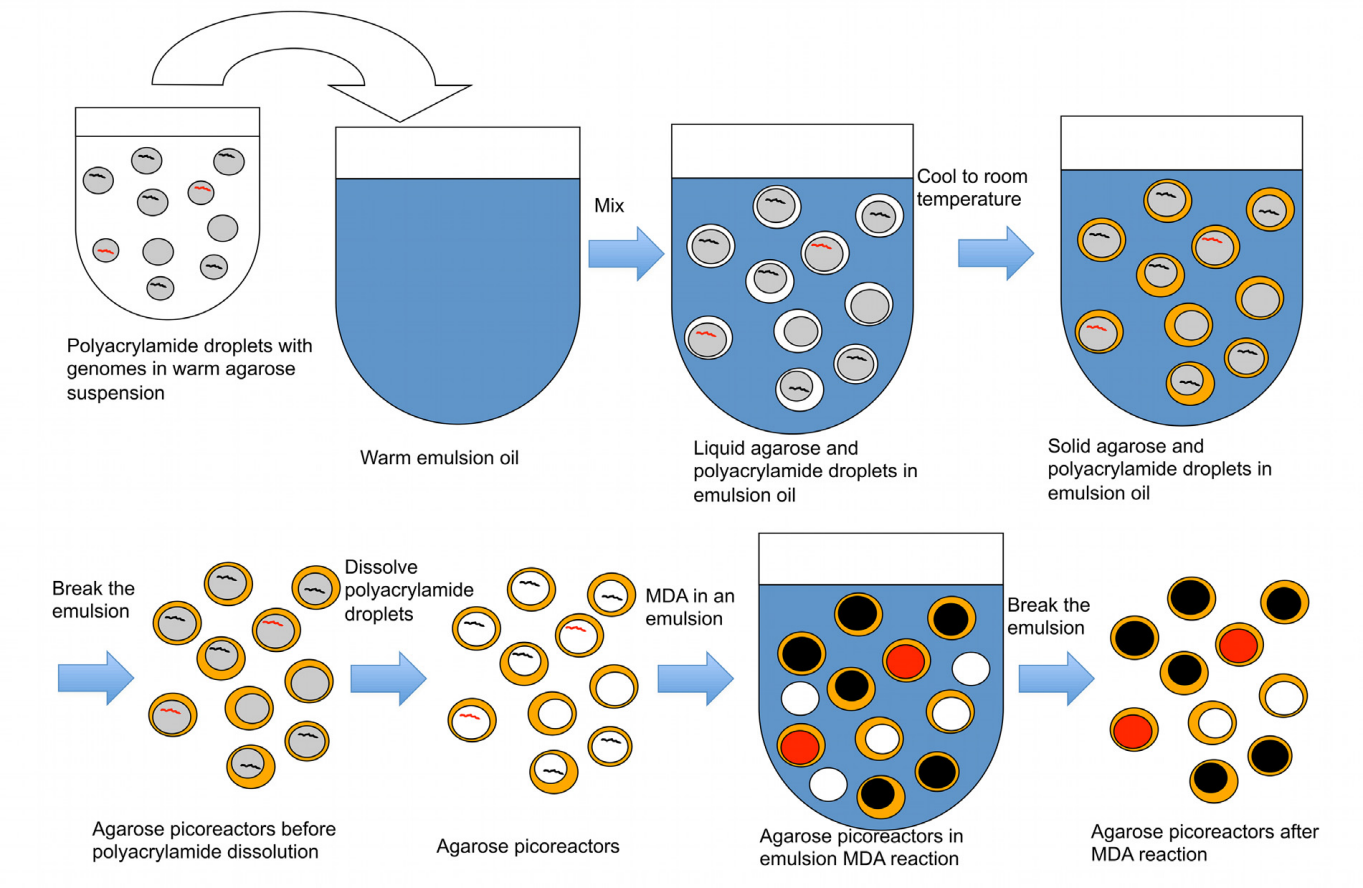
**A procedure to create agarose picoreactors for single-genome amplification.** An agarose layer is added onto polyacrylamide droplets that contain individual genomes (**Figures 1** and **2**). Multiple displacement amplification (MDA) is performed in an emulsion to ensure individual amplification of each genome. Black lines and black-filled circles represent target-less unamplified and amplified genomes, respectively. Red lines and red-filled circles represent target-containing unamplified and amplified genomes, respectively. White-filled circles represent empty picoreactors.

**FIGURE 4:**
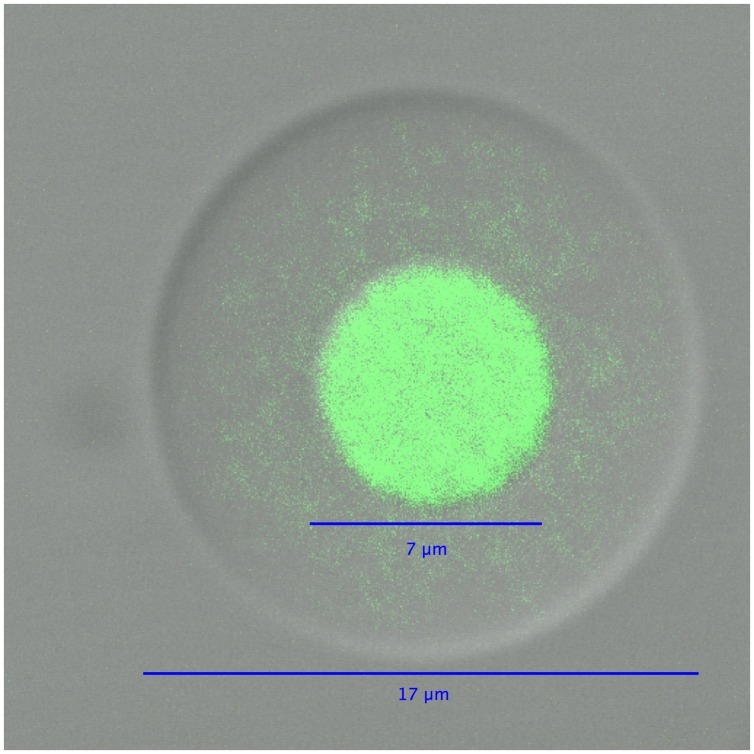
**A differential interference contrast/confocal micrograph of an agarose picoreactor after genome amplification in an emulsion MDA reaction.** The green fluorescence of SYBR Green dye is used to visualize DNA.

In the second part of the technique, an agarose layer is added to the polyacrylamide droplets, which are subsequently dissolved prior to performing MDA reactions (**Figure 3**). The polyacrylamide must be dissolved because its polymer structure is too dense to allow an efficient MDA reaction. Dissolving the polyacrylamide droplet yields microbial genomes in picoliter liquid volumes within an agarose layer. These agarose picoreactors are mixed with reagents for the MDA reaction and then mixed into an emulsion. Agarose is permeable to enzymes and small molecules but not to genomic or amplified DNA (**Figure 4**). Because the only DNA in the agarose picoreactors comes from single-genomes trapped in the polyacrylamide droplets, the picoreactors provide a relatively sterile environment for the MDA reaction. The sterility of the reaction is also enhanced by performing the MDA reaction in an emulsion in which each picoreactor occupies an individual reaction compartment. Moreover, the contamination can be further decreased by UV treatment of the MDA reagents (Woyke et al., 2011).

### Labeling Picoreactors Containing Genomes with a Target Gene

**FIGURE 5:**
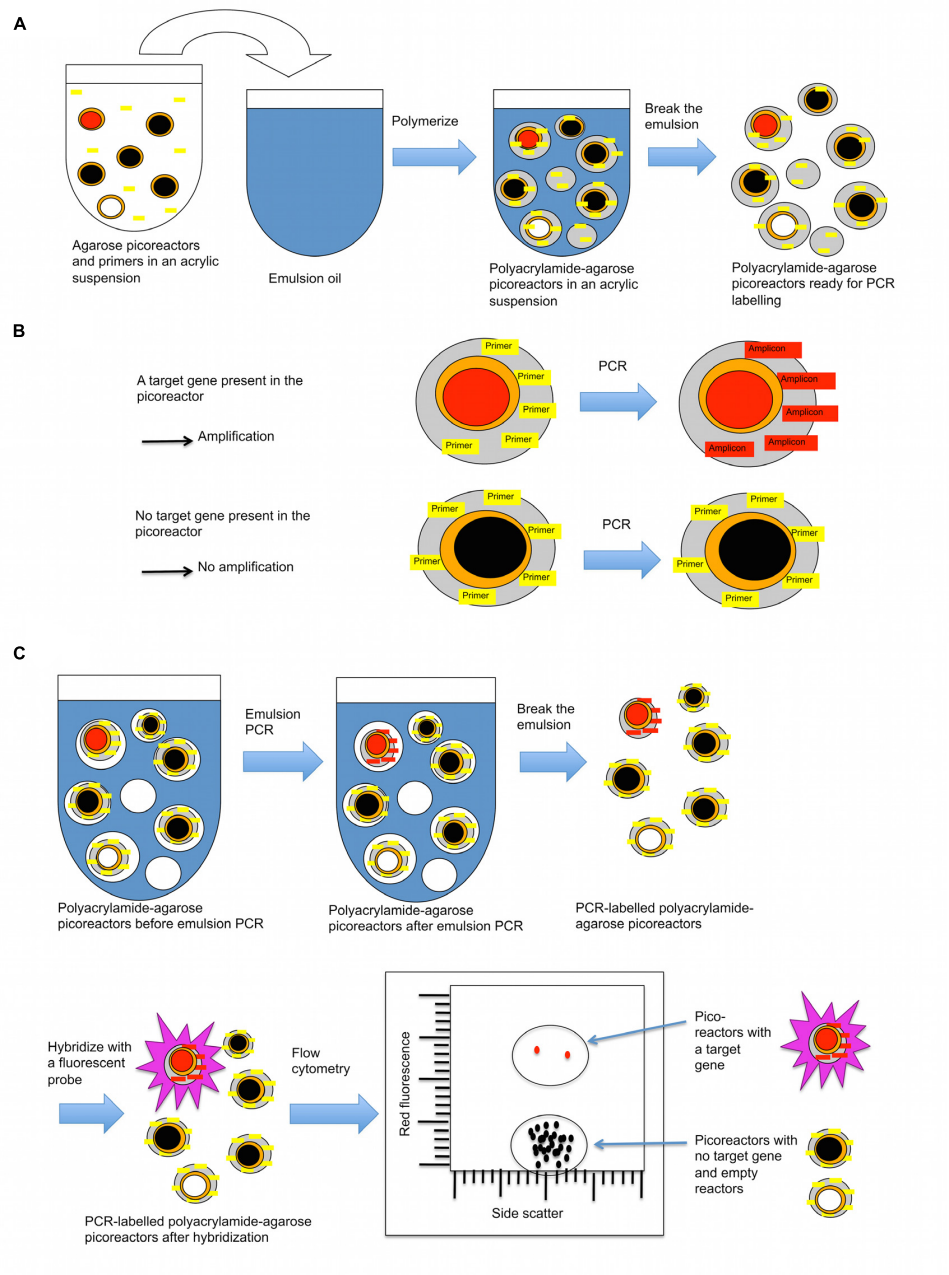
**A layer of polyacrylamide is added to picoreactors to prepare them for PCR-based labeling. (A)** Procedure for adding a polyacrylamide layer onto agarose picoreactors (prepared in **Figure 3**). The acrylamide suspension contains an acrydite-modified primer that becomes covalently attached to the polyacrylamide matrix during polymerization. **(B)** The polyacrylamide matrix contains a covalently attached primer complementary to the target gene. If the amplified genome in the picoreactor contains the target gene of interest (red-filled circle), an amplicon is synthesized by PCR that remains covalently attached to the polyacrylamide matrix. For droplets with no target gene of interest (black-filled circle, picoreactors containing genomes with no target gene; white-filled circle, empty picoreactors), no amplicon is generated by PCR. Agarose residues (orange) melt during PCR and do not interfere with the reaction. **(C)** After PCR, the droplets with attached amplicons are labeled using a complementary fluorescent probe. The labeled droplets are then differentiated by their increased fluorescence using a flow cytometer.

In the third part of the technique, a new layer of polyacrylamide containing immobilized PCR primers complementary to the target gene is added to the picoreactors (**Figure 5**). The picoreactors are then mixed with PCR reagents to form an emulsion, and the target gene is amplified by PCR. PCR can be performed on the picoreactors because the new layer of polyacrylamide provides a matrix that supports the DNA generated by the MDA reaction and structurally reinforces the picoreactors, conferring heat-tolerance to them. The agarose in the picoreactors melts and does not interfere with the PCR reaction (Mak et al., 2008). Target gene amplicons accumulate in the polyacrylamide matrix of the droplet where they are hybridized with fluorescent probes, thus labeling the picoreactors. Labeled picoreactors can subsequently be separated from empty picoreactors and picoreactors not containing the target gene using flow cytometry. The labeling is highly specific to the target gene because a fluorescent signal requires both successful PCR amplification and successful hybridization to the target amplicon. The amplification is done using deoxyuridine instead of deoxythymidine to permit amplicon degradation by uracil-specific excision reagents after the flow cytometric selection.

### Flow-Cytometric Validation of the Method

**FIGURE 6:**
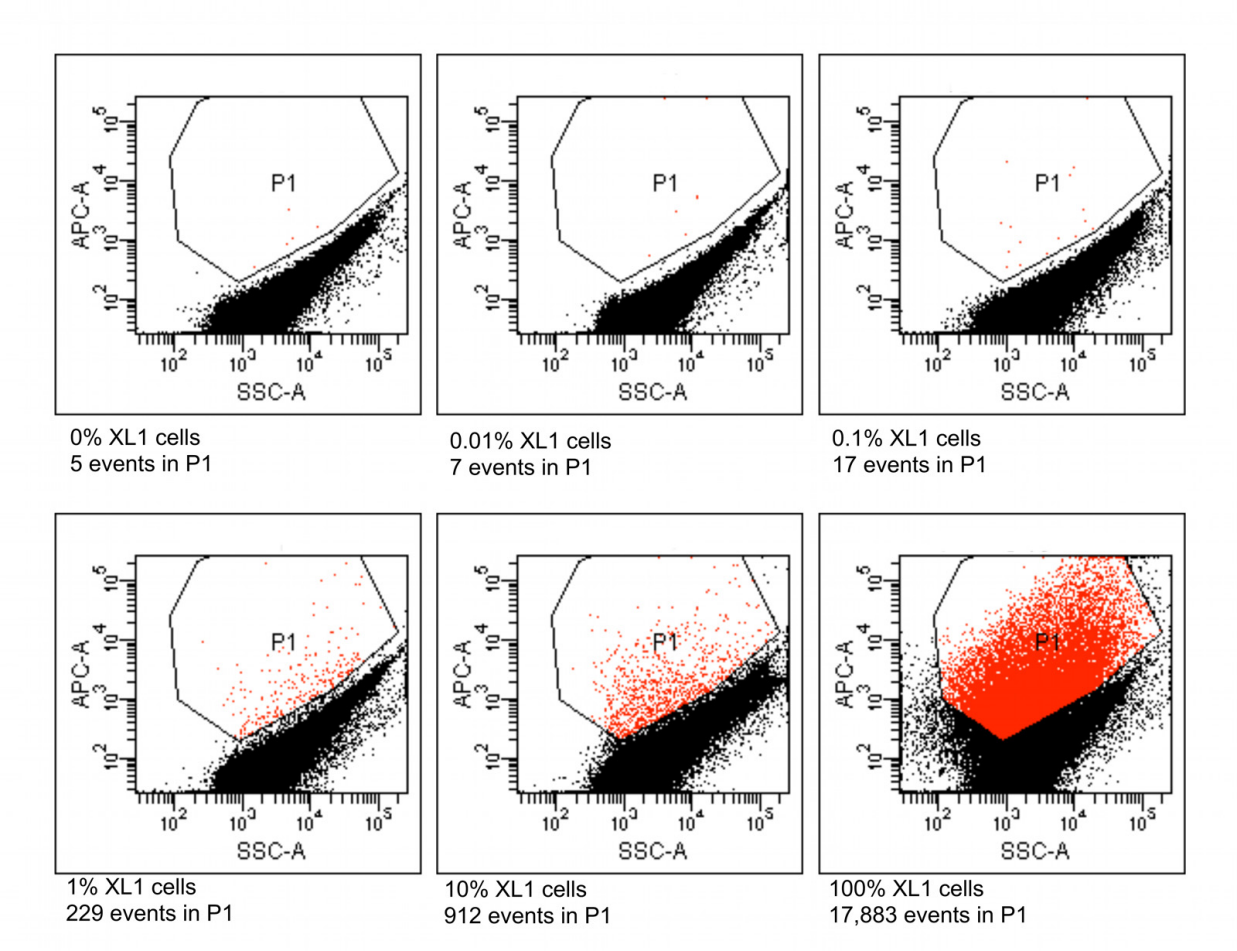
**Flow cytometric results from picoreactors of different suspensions of *E. coli* XL1 and *E. coli* MC1061.** The *E. coli* XL1 genome contains a single copy of a tetracycline resistance gene, whereas the *E. coli* MC1061 genome contains none. The genomes are amplified in agarose picoreactors and labeled by emulsion PCR and fluorescent probe hybridization targeting the tetracycline resistance gene. Picoreactors containing the XL1 genome exhibit increased red fluorescence. The parameter SSC-A on the *x*-axis refers to a side-scatter value that correlates with the light-scattering property of the analyzed particles. The parameter APC-A on the *y*-axes refers to the intensity of red fluorescence. Events in the P1 gate are labeled picoreactors containing XL1 genomes and therefore have increased red fluorescence. Altogether, 100,000 events were collected from each suspension. The fluorescent events in a suspension containing no XL1 cells (0%) are false-positive events. The non-fluorescent events in suspensions containing 100% XL1 cells represent empty picoreactors.

**FIGURE 7:**
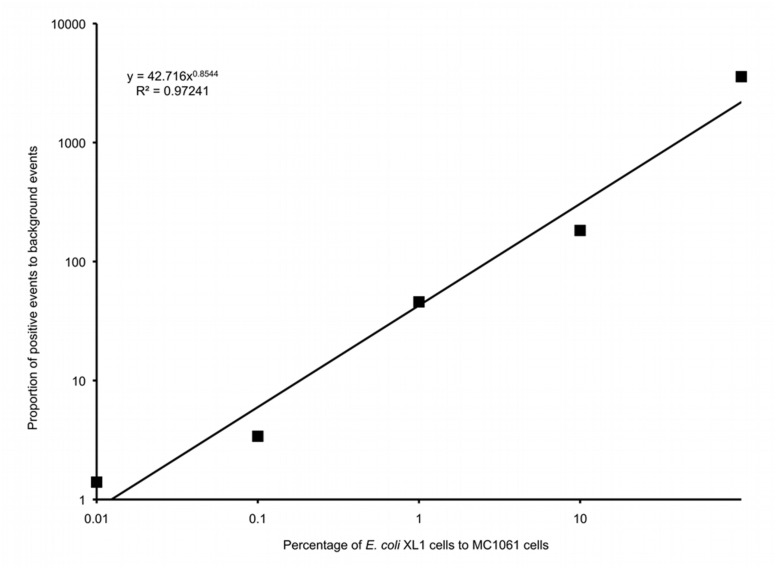
**A logarithmic plot of the proportion of false positive background events to positive events in gate P1 versus the percentage of *E. coli* XL1 to *E. coli* MC1061 in the initial cell suspension**.

Six different suspensions of *E. coli* XL1 and *E. coli* MC1061 cells were prepared in which the percentage of *E. coli* XL1 ranged from 0 to 100% of the total. The genome of *E. coli* XL1 contains a tetracycline resistance gene, whereas the genome of *E. coli* MC1061 does not. The cells were processed as described above to amplify the genomes and label them based on the presence of the tetracycline resistance gene. When analyzed using a flow cytometer, picoreactors carrying the *E. coli* XL1-genome appeared as bright fluorescent events (**Figure 6**). The less-fluorescent events correspond to empty picoreactors and picoreactors containing the *E. coli* MC1061 genome. Altogether, 100,000 events were collected for each suspension. The proportion of *E. coli* XL1 to MC1061 cells remained similar between the initial cell suspension and the labeled picoreactors. Based on the scatterplots, as little as 0.1% *E. coli* XL1 cells in the initial cell suspension (relative to MC1061 cells) could be differentiated using the method. The number of fluorescent events correspond to the percentage of XL1 cells (power equation *R*^2^-value 0.97; **Figure 7**).

### Flow-Cytometric Application of the Method to a Spiked Environmental Sample

**FIGURE 8:**
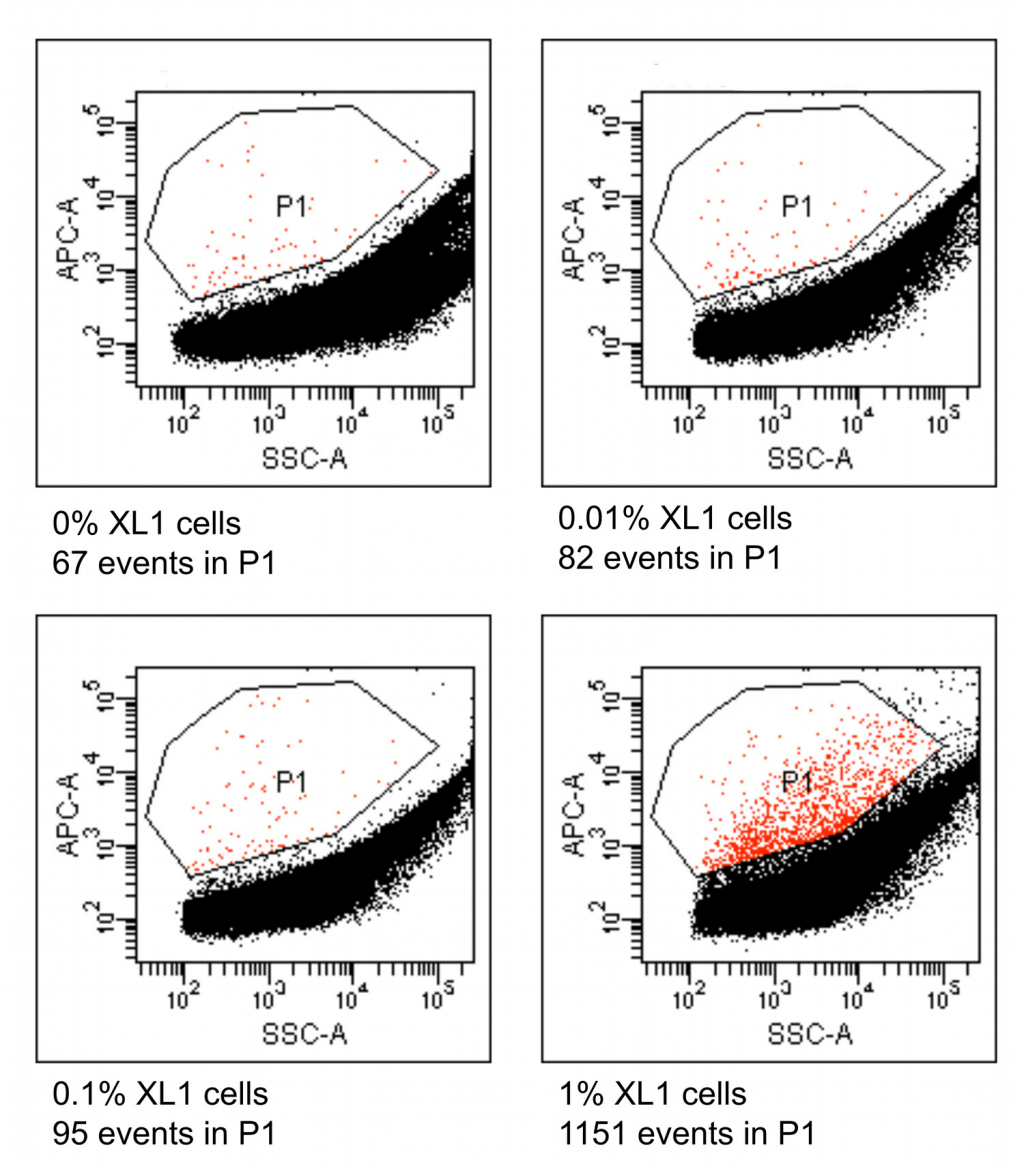
**Flow cytometric results from picoreactors of different suspensions of *E. coli* XL1 and microbes extracted from marine sediment.** The *E. coli* XL1 genome contains a single copy of a tetracycline resistance gene, whereas the sediment microbes do not contain the gene. The genomes are amplified in agarose picoreactors and labeled by emulsion PCR and fluorescent probe hybridization targeting the tetracycline resistance gene. Picoreactors containing the XL1 genome exhibit increased red fluorescence. The parameter SSC-A on the *x*-axis refers to a side-scatter value that correlates with the light-scattering property of the analyzed particles. The parameter APC-A on the *y*-axes refers to the intensity of red fluorescence. Events in the P1 gate are labeled picoreactors containing XL1 genomes and therefore have increased red fluorescence. Altogether, 100,000 events were collected from each suspension. The sediment microbes exhibit a higher rate of false positive events than the mixture of the two *E. coli* strains (**Figure 6**), as indicated by the suspension containing no XL1 cells (0%).

Four different suspensions of *E. coli* XL1 and diverse microbial cells extracted from marine sediment were prepared where the percentage of *E. coli* XL1 ranged from 0 to 1% of the total cells. The genome of *E. coli* XL1 contains a tetracycline resistance gene, whereas the sediment microbes do not, according a PCR reaction using isolated sediment DNA as a template (data not shown). The cells were processed as described above to amplify the genomes and label them based on the presence of the tetracycline resistance gene. When analyzed using a flow cytometer, picoreactors carrying the *E. coli* XL1-genome appeared as bright fluorescent (**Figure 8**). The less-fluorescent events correspond to empty picoreactors and picoreactors containing other genomes. Altogether, 100,000 events were collected for each suspension. The number of false positive background events is higher with an environmental sample than with the mixture of two *E. coli* strains. Nevertheless, mixtures with 0.1 and 1% of *E. coli* XL1 exhibited an elevated amount of fluorescent events. With the observed frequency of false positive events, target proportions of 0.01, 0.1 and 1% could be identified with respective false positive rates of 8 false to 2 true, 6 false to 4 true, and 1 false to 16 true. The false positive background events appear even when a fluorescent probe has not been hybridized to the picoreactors and are most likely autofluorescent cells or mineral particles.

## Discussion

Emulsions are an inexpensive and simple way to divide chemical or enzymatic reactions into millions of parallel reactions. This property of emulsions have been successfully exploited for a number of applications, including pyrosequencing technology, and BEAMing (Margulies et al., 2005; Diehl et al., 2006). Preparing a cell suspension by chemical and mechanical detachment of microbes from biofilm structures and filaments allows an emulsion approach to be used to cast polymer shells on a large quantity of cells without bias from cell morphology (Zengler et al., 2002). Here, polyacrylamide was polymerized on microbial cells in an emulsion to construct a support matrix for the genomic material. This is necessary because some cells are highly resistant to cell lysis, whereas others lyse completely even after a brief enzymatic treatment. The technique presented in this study allows extended incubation times with high concentrations of lytic enzymes because it supports genomic DNA as a discreet package, even after the cell wall and other structures have been completely degraded. Therefore, this method can be used to lyse all cell types, from fragile to highly resistant, in the same reaction. Although the model organism in this study was a Gram-negative bacterium, we expect that the method is equally applicable to different bacterial, archaeal, and eukaryotic cells. Traditionally, the different lysis reaction requirements for different cell types and the risk of complete cell lysis and genome dispersion have posed major difficulties for methods such as CARD-FISH and *in situ*-PCR, which also rely on exposing genomic DNA to enzymes (Hodson et al., 1995; Kubota et al., 2008).

The polyacrylamide support matrix was constructed using a cross-linker ontaining a disulfide bond that can effectively be cleaved in mild reducing conditions, such as in an MDA reaction. Such crosslinker has been used previously to permanently reshape polyacrylamide gels by consequtive reduction, reshaping and oxidation steps (Greytak et al., 2001). This study presents the first demonstration of the use of such material to construct scaffoldings for miniaturized reactors for parallel MDA reactions. Agarose was chosen as the material for the reactor walls because of its sufficient porosity to permit reagent diffusion and its inertness in PCR reactions (Mak et al., 2008). The agarose picoreactors permit the MDA reactions to be performed in an emulsion to avoid any cross-contamination between individual reactors. After the MDA reaction, a second layer of polyacrylamide was prepared using the disulfide bond-containing crosslinker and an 5′-acrydite-modified primer that becomes covalently attached to the polyacrylamide matrix. Acrydite-modified primers have previously been used to provide covalent attachment of primers and amplicons to polyacrylamide support matrix in, e.g., polony sequencing technology (Shendure et al., 2005). To our knowledge, covalently bound amplicons have not previously been used as targets for FISH probes.

The screening procedure is sensitive enough to detect microbial genomes in which the target gene is present in as little 0.1% of the total initial cell population. The method performs well on complex sediment microbial population spiked with low amounts of *E. coli* XL1, despite the rate of false positive events that increases with dilution. Until now, such target-gene screening of genomes has been done by testing single amplified genomes on 96-well plates by PCR (Stepanauskas and Sieracki, 2007), an approach that would require screening an average of 10 plates to find one target genome with a target-gene frequency of 0.1%. Clearly, a high-throughput screening procedure such as that described here would be invaluable for studying microbes that are not predominant community members.

Multiple displacement amplification reaction is known to function in nanoliter volumes in an efficiency comparable to a standard reaction volume of microliters (Marcy et al., 2007). Although we could observe DNA amplification by MDA also in picoliter-volume, we cannot currently draw conclusion about the efficiency of the MDA reaction. We envision that in future applications, the MDA-amplified DNA could be re-amplified and used for sequencing applications. This would require circumventing the paraformaldehyde fixation of the cells and using a different polymer chemistry that does not cause DNA damage such as PEG-acrylates (Chung and Deisseroth, 2013).

In the present state of the technique, the combination of of *in situ* PCR and FISH to detect low copy genomic targets in rare members of highly diverse cell populations will permit entirely novel experimental possibilities in microbiology and metagenomics.

## Author Contributions

MT and MV designed research; MT performed research; MT analyzed data; MT and MV wrote the paper.
